# Maternal imbalance between pro-angiogenic and anti-angiogenic factors in HIV-infected women with pre-eclampsia

**DOI:** 10.5830/CVJA-2013-029

**Published:** 2013-06

**Authors:** Nalini Govender, Thajasvarie Naicker, Jagidesa Moodley

**Affiliations:** Optics and Imaging Centre, University of KwaZulu-Natal, South Africa; Optics and Imaging Centre, University of KwaZulu-Natal, South Africa; Women’s Health and HIV Research Group, University of KwaZulu-Natal, South Africa

**Keywords:** sFlt1, pre-eclampsia, anti-angiogenic factors, HIV

## Abstract

**Abstract:**

Angiogenic imbalance contributes to the development of preeclampsia. We evaluated the protein expression of the proangiogenic placental growth factor (PlGF) and transforming growth factor beta 1 (TGF-β_1_) compared with the anti-angiogenic soluble fms-like tyrosine kinase receptor (sFlt1) and soluble endoglin (sEng) in HIV-infected normotensive and pre-eclamptic pregnancies.

Blood was obtained from 110 pregnant women, enrolled in four groups, namely, HIV-negative normotensives (27); HIV-positive normotensives (31); HIV-negative pre-eclamptics (27) and HIV-positive pre-eclamptics (25), and was used to measure PlGF, TGF-β1, sFlt1 and sEng levels.

Increased sFlt1 and sEng levels were associated with the pre-eclamptics (HIV negative and positive) compared with their counterparts. Decreased PlGF levels were observed between the HIV-negative pre-eclamptics versus HIV-negative normotensives, but levels differed significantly (*p* = 0.02) among the normotensives (HIV negative and positive). TGF-β_1_ remained unchanged across all groups. Higher sEng/TGF-β_1_ ratios were associated with the pre-eclamptics (HIV negative and positive) compared with their counterparts. This study demonstrated increased sFlt1 and sEng levels in pre-eclamptic compared with normotensive pregnancies, irrespective of the HIV status.

## Abstract

Pre-eclampsia, a clinical syndrome unique to human pregnancy is characterised by new-onset hypertension and proteinuria, which present after the 20th week of gestation.^1^-^4^ Although several studies in the last few decades have investigated the pathogenesis of this disorder, limited progress has been made in establishing the exact cause.^5^,^6^

Currently, pre-eclampsia is reported to be a two-stage disorder, namely, a pre-clinical/asymptomatic, and a clinical stage.^2^,^7^ The first stage is characterised by abnormal placentation leading to a hypoxic placenta, oxidative stress and immune dysregulation, while the second stage is characterised by the placental discharge of soluble factors, such as sFlt1 and sEng into the maternal circulation, resulting in widespread endothelial dysfunction and the clinical syndrome of hypertension, proteinuria, intrauterine growth restriction (IUGR) and thrombocytopenia.^2^,^7^-^10^

Both pre-eclampsia and HIV infection are common conditions in sub-Saharan Africa and major causes of maternal deaths.^11^ Recent studies have reported that the persistent infection of HIV-infected individuals contributes to the development of chronic arterial injury and subsequent endothelial damage, atherosclerosis and thrombosis.^12^ Furthermore, untreated HIV-infected patients may be prone to endothelial dysfunction.^12^ HIV infection also seems to affect the mechanisms implicated in the aetiology of pre-eclampsia and IUGR.

Normal pregnancy is characterised by an altered immune sensitivity, thereby allowing maternal resistance against any infection and foetal tolerance, while pre-eclampsia is a hyperactive immune response.^13^,^14^ It is plausible that the immune insufficiency stimulated by HIV together with the normal immune changes of pregnancy may reduce a predisposition to the immune hyper-reactivity that is associated with pre-eclampsia.^15^-^17^ Therefore it is not surprising that some reports have shown that a reduced rate of pre-eclampsia prevails among untreated HIV-infected patients in comparison with those on highly active antiretroviral therapy (HAART).^14^,^18^

The administration of HAART is reported to enhance maternal immune reconstitution by re-establishing the mother’s immune response to foetal antigens, and consequently making the woman susceptible to the development of pre-eclampsia.^14^ Conflicting reports however, have created uncertainty as to whether HIV-infected pregnant women on HAART have lower rates of pre-eclampsia.^17^-^19^ This uncertainty may impact on both maternal and perinatal morbidity and mortality in a geographical region with a high prevalence of HIV.

Angiogenic biomarkers have been suggested for the early detection of pre-eclampsia in high-income countries, despite the lack of robust evidence for their use.^4^,^17^ Therefore, this study set out to examine the role of pro- and anti-angiogenic factors in the aetiology of pre-eclampsia in a setting of high rates of HIV infection.

## Methods

Institutional ethical and regulatory approvals were obtained. Clinical characteristics such as maternal and gestational age, parity, maternal, baby and placental weight, and blood pressure were collected during antenatal recruitment.

Venous blood samples were collected from 110 pregnant black African women at term attending a tertiary maternity unit in Durban, KwaZulu-Natal, South Africa. All blood samples were centrifuged within two hours at 3 500 rpm for 10 min at 4°C. Serum aliquots were then carefully transferred into new tubes and stored at –70°C until analysis. Enzyme-linked immunoassays for PlGF (1:2), TGF-β1 (1:40), sFlt1 (1:5) and sEng (1:5) were performed in triplicate according to the manufacturer’s instructions (R&D Systems, Minneapolis, MN).

Fifty-two of the blood samples were obtained from pre-eclamptics and 58 from normotensive pregnant women. These groups were further subdivided into HIV-negative and HIV-positive sub-groups.

Inclusion criteria for pre-eclampsia were persistent systolic blood pressure 140 mmHg and diastolic blood pressure 90 mmHg, taken at least six hours apart, after 20 weeks’ gestation in a previously normotensive patient. Proteinuria was defined as urine protein concentration of ≥ 300 mg/dl or 1+ on a urine dipstick in at least two random specimens collected at least four hours apart.

Exclusion criteria for all groups was chorio-amnionitis, chronic hypertension, eclampsia and abruptio placentae; intrauterine death, pre-gestational diabetes, gestational diabetes and chronic renal disease; systemic lupus erythematous, sickle cell disease and anti-phospholipid antibody syndrome; thyroid disease, cardiac disease and active asthma requiring medication during pregnancy and pre-existing seizure disorders.

## Statistical analysis

Inter-group analysis was conducted using the non-parametric Kruskal-Wallis test. Descriptive statistics were utilised and outcome variables are presented as median (interquartile range). Where differences were found in the Kruskal-Wallis test, Dunn’s *post hoc* test was used for multiple comparisons. A probability level of *p* < 0.05 was considered statistically significant. All statistical analyses were conducted using GraphPad Prism® version 5.01.

## Results

Clinical characteristics for the pre-eclamptic and normotensive participants (*n* = 110) were divided into HIV-positive (*n* = 56) and HIV-negative groups (*n* = 54), respectively, namely, (1) HIV-negative normotensive (N–): BP ≤ 120/80 mmHg (*n* = 27); (2) HIV-positive normotensive (N+): BP ≤ 120/80 mmHg; CD_4_ < 200 cells/μl (*n* = 31); (3) HIV-negative pre-eclamptic (P–): BP 140/90 mmHg (*n* = 27) and (4) HIV-positive pre-eclamptic (P+): BP 140/90 mmHg; CD_4_ < 200 cells/μl (*n* = 25) (Table 1).

**Table 1.** Demographic And Clinical Profile Of Patients Recruited For Immunoassays

	*Normotensive pregnant women (N–)*	*HIV normotensive pregnant women (N+)*	*Pre-eclamptic pregnant women (P–)*	*HIV pre-eclamptic pregnant women (P+)*	p*-value*
Number	27	31	27	25	
Age (years)	24 (21–26)	27 (24–30)	25 (20–32)	32 (25.5–34)	0.0009*
Gestational age (weeks)	38 (38–39)	39 (38–40)	38 (37–40)	38 (36–38)	0.0026*
Parity	0 (0–1)*	1 (1–2)	1 (0–1)	1 (0.5–3)	0.0174*
Maternal weight (kg)	66 (59–74)	74 (65–82)	75 (65–96)	82 (64–106)	0.0321*
Birth weight (kg)	3.2 (3–3.4)	3.4 (3–3.7)	3.2 (2.6–3.8)	2.9 (2.7–3.4)	ns
Placental weight (g)	360 (300–400)	470 (380–500)	480 (430–510)	480 (380–515)	< 0.0001*
Systolic BP (mmHg)	110 (108–115)	112 (107–120)	154 (150–162)	150 (145.5–159)	< 0.0001*
Diastolic BP (mmHg)	70 (67–73)	70 (67–74)	94 (90–104)	9 6 (87–99.5)	< 0.0001*

Medians (range) are presented; Kruskal-Wallis test and the post hoc Dunns multiple comparison test was used for statistical analysis, *n* = 110. *p < 0.05

A significant difference was detected for maternal and gestational age, parity, maternal and placental weight, and systolic and diastolic blood pressure (*p* < 0.05) between the four groups (Kruskal-Wallis test, Table 1). Mean maternal age ranged between 23 and 30 years while the mean gestational age ranged between 37 and 39 weeks (Table 1).

For maternal weight, the Kruskal-Wallis test showed an overall significance (*p* < 0.05). The Dunn’s multiple comparison tests identified a significant difference between only the HIV-positive pre-eclamptic and the HIV-negative normotensive pregnant women (*p* = 0.0321; Table 1). However, for placental weight (Table 1), a significant difference was evident between the HIV-positive pre-eclamptic and HIV-negative normotensive pregnant women (*p* < 0.0001), the HIV-negative pre-eclamptic and HIV-negative normotensive pregnant women (*p* < 0.0001) and the HIV-positive normotensive and HIV-negative normotensive pregnant women (*p* < 0.0001; Table 1).

For systolic blood pressure (Table 1), a significant difference was evident between the HIV-positive pre-eclamptic and HIV-negative normotensive pregnant women (*p* < 0.0001), the HIV-positive pre-eclamptic and the HIV-positive normotensive pregnant women (*p* < 0.0001), the HIV-negative pre-eclamptic and the HIV-negative normotensive pregnant women (*p* < 0.0001) and the HIV-negative pre-eclamptic and HIV-positive normotensive pregnant women (*p* < 0.0001). A similar pattern was observed for diastolic blood pressure, as indicated in Table 1.

## Pro-angiogenic and anti-angiogenic factors

**Fig. 1.**
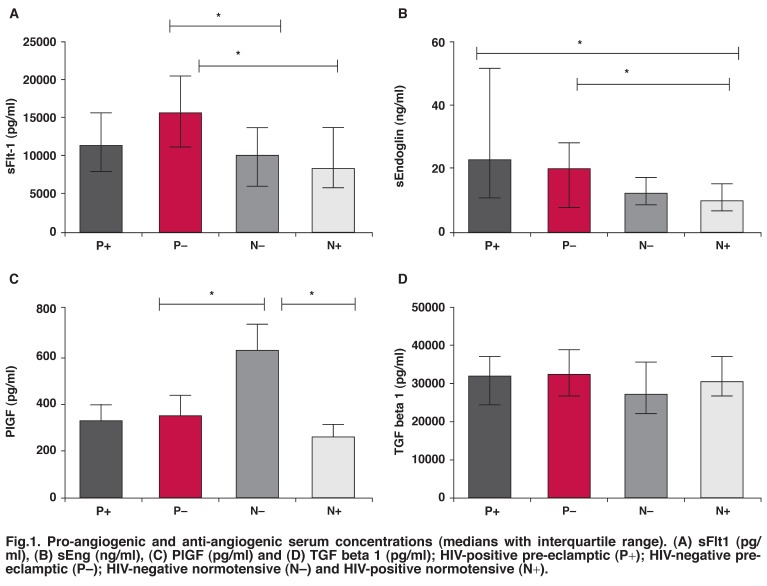
Pro-angiogenic and anti-angiogenic serum concentrations (medians with interquartile range). (A) sFlt1 (pg/ml), (B) sEng (ng/ml), (C) PlGF (pg/ml) and (D) TGF beta 1 (pg/ml); HIV-positive pre-eclamptic (P+); HIV-negative preeclamptic (P–); HIV-negative normotensive (N–) and HIV-positive normotensive (N+).

**Fig. 2.**
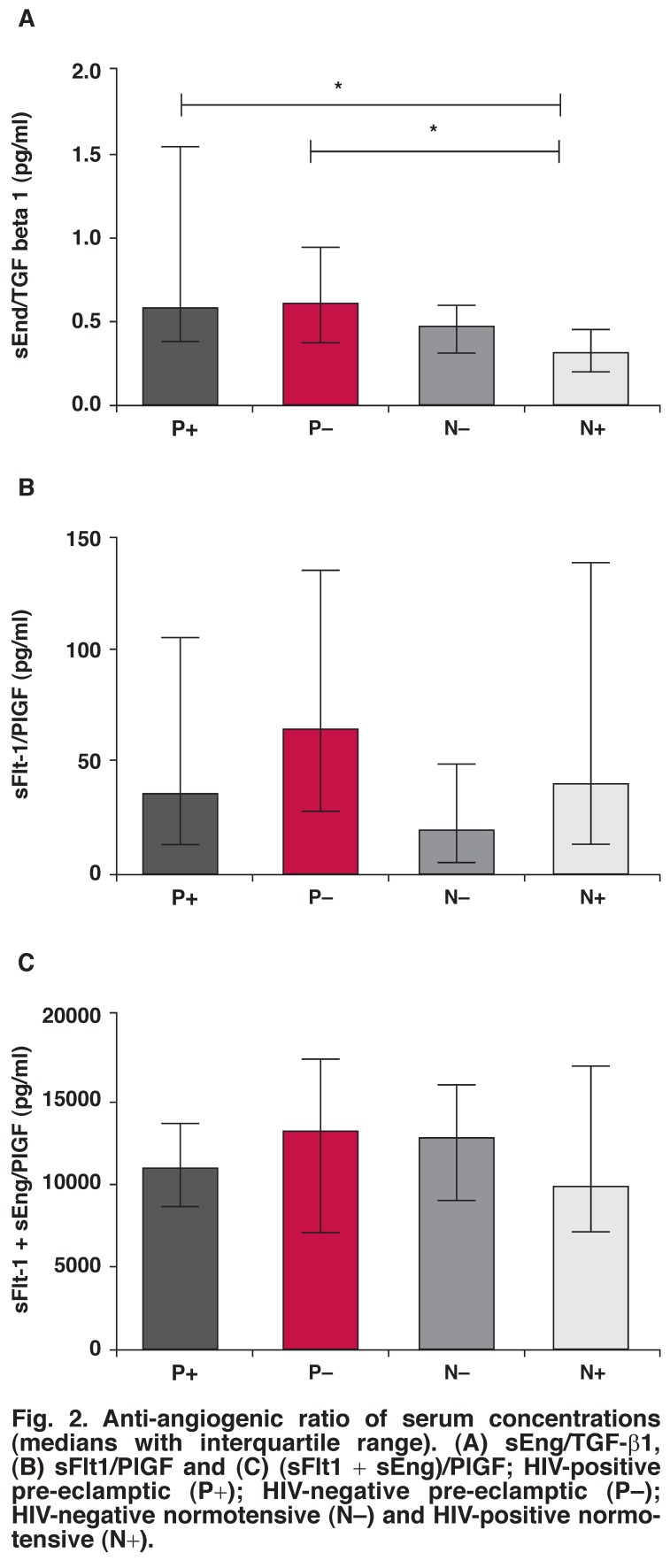
Anti-angiogenic ratio of serum concentrations (medians with interquartile range). (A) sEng/TGF-β1, (B) sFlt1/PlGF and (C) (sFlt1 + sEng)/PlGF; HIV-positive pre-eclamptic (P+); HIV -negative pre-eclamptic (P–); HIV-negative normotensive (N–) and HIV-positive normotensive (N+).

Serum concentrations for all evaluated pro-angiogenic (PlGF and TGF-β1) and anti-angiogenic (sFlt1 and sEng) factors varied (Table 2, Figs 1a–d and 2a–c). A significant difference was observed for sFlt1, sEng and PlGF (*p* < 0.05) between the groups (Figs 1a–d). For sFlt1, the Kruskal-Wallis test showed an overall significance (*p* < 0.05). The Dunn’s multiple comparison test revealed a significant difference between HIV-negative pre-eclamptic and HIV-negative normotensive pregnant women (*p* = 0.0061), and HIV-negative pre-eclamptic and HIV-positive normotensive pregnant women (*p* = 0.0061).

**Table 2.** Comparison Of Pro-Angiogenic And Anti-Angiogenic Factors Of Maternal Serum Across Study Groups

	*Normotensive pregnant women (N–)*	*HIV normotensive pregnant women (N+)*	*Pre-eclamptic pregnant women (P–)*	*HIV pre-eclamptic pregnant women (P+)*	p*-value*
Number	27	31	27	25	
sFlt-1(pg/ml)	10249 (6308–13708)	8578 (5898–13769)	15617 (11257–20641)	11494 (8203–15784)	0.006*
sEng (ng/ml)	13.14 (9.6–17.92)	10.4 (7.3–16)	20.5 (8.5–29)	23 (11.8–52.5)	0.002*
PlGF (pg/ml)	488.6 (183.9–848.3)	207.1 (102.6–358.6)	202.2 (47.9–490.4)	229.3 (74.3–615.9)	0.021*
TGF-β1 (pg/ml)	27640 (22308–35771)	31164 (26916–37474)	32652 (27295–39328)	32301 (24983–37355)	ns
sEng/TGF-β1 (pg/ml)	0.48 (0.33–0.6)	0.32 (0.2–0.45)	0.62 (0.38–0.95)	0.59 (0.38–1.5)	0.002*
sFlt-1/PlGF (pg/ml)	21.4 (7.2–50.8)	41.6 (14.5–140.7)	66.5 (28.3–136.9)	37.3 (14.8–106.2)	ns
sFlt1+sEng/ PlGF (pg/ml)	12392 (8792–15700)	9422 (7014–16914)	12988 (6859–17276)	10772 (8345–13527)	ns

Medians (range) are presented; Kruskal-Wallis test and the post hoc Dunns multiple comparison test was used for statistical analysis, *n* = 110. *p < 0.05; non-significant (ns).

A significant difference for sEng (Table 2, Fig. 1) was evident between HIV-positive pre-eclamptic and HIV-positive normotensive pregnant women (*p* = 0.0017), and HIV-negative pre-eclamptic and HIV-positive normotensive pregnant women (*p* = 0.0017). Likewise for PlGF, a significant difference was found between the HIV-negative pre-eclamptic and HIV-negative normotensive pregnant women (*p* = 0.021), and the HIV-negative normotensive and HIV-positive normotensive pregnant women (*p* = 0.021). However, TGF-β_1_ did not differ significantly between groups (*p* = 0.359; Fig. 1).

Anti-angiogenic ratio analyses revealed a significant difference only for sEng/TGF-β_1_ ratios (*p* < 0.05, Table 2, Fig. 2a). Accordingly, the Dunn’s multiple comparison test revealed a significant difference for both HIV-positive pre-eclamptic and HIV-positive normotensive pregnant women (*p* = 0.002), and HIV-negative pre-eclamptic and HIV-positive normotensive pregnant women (*p* = 0.002).

## Discussion

Since placental delivery is the only cure for pre-eclampsia, its clinical management is dependent on gestational age and disease severity. In our study, gestational age and placental weight varied among the study groups. The pre-eclamptic groups (HIV positive and negative) delivered at a slightly earlier gestational period compared with the normotensive groups.

Our data revealed that placental weights for the pre-eclamptic groups were greater than the normotensive groups. This is surprising as one would have expected the placental weights in pre-eclamptics to be lower in view of the fact that the pre-eclamptics delivered at a lesser gestational age and the fact that pre-eclampsia is associated with IUGR. Alternatively, this higher placental weight may be attributed to the late onset of pre-eclampsia.

Although we did not correlate foetal growth with gestational age following delivery, there is circumstantial evidence that women with gestational hypertension and mild pre-eclampsia tend to have slightly bigger babies and larger placental masses than their normotensive counterparts at birth. It is plausible that mild increases in blood pressure could cause a concomitant increase in placental perfusion pressure and increased oxygen supply, resulting in increased placental size.

However, a recent epidemiological analysis conducted by Eskild and Vatten (2010) reported conflicting data with regard to placental weight.^20^ Pre-eclampsia is hypothesised to be due to placental dysfunction, however these investigators have suggested that placental weight may not be a risk indicator for the placental dysfunction evident in pre-eclampsia. In addition, the placenta is identified as the major angiogenic contributor, and that the imbalance evident in pre-eclampsia may be associated with placental hypoxia.^20^

Therefore the pre-eclamptic placenta is involved with the cause of the disease and is implicated in the production of elevated levels of sFlt1 and sEng.^2^,^21^-^23^ This elevation is believed to disrupt the balance of the pro-angiogenic factors, thereby decreasing their bioavailability, with the subsequent vascular maladaptation of pre-eclampsia.

Our study further demonstrated variations between the pro-angiogenic (PlGF and TGF-β_1_) and anti-angiogenic factors (sFlt1 and sEng) that occurred in pre-eclamptic (HIV negative and positive) and normotensive (HIV negative and positive) pregnancies, lending credence to the anti-angiogenic theory of pre-eclampsia. To our knowledge, there are no available data that explore the relationship of HIV with circulating pro-angiogenic and anti-angiogenic factors in pre-eclampsia.

VEGF is recognised as a powerful endothelial-specific mitogen and its significant role in angiogenesis is well documented.^24^-^26^ It is functional through the two high-affinity receptor tyrosine kinases VEGFR1 (Flt1) and VEGFR2 (Flk1). PlGF is also a member of the VEGF family, which binds to Flt1, thereby supplementing the pro-angiogenic effects of VEGF.^24^-^26^ However, a soluble isoform and a splice variant of Flt1 have been identified as sFlt, which contains a ligand-binding domain but lacks a trans-membrane and cytoplasmic domain.^27^ Karumanchi and co-workers further demonstrated an excess production of sFlt1 by the pre-eclamptic placental trophoblasts and subsequent discharge into the maternal circulation, implicating it as a key role player in the aetiology of this maternal syndrome.^27^

Transforming growth factor-beta (TGF-β_1_), comprising three isoforms, is important for the development of the embryo, inflammation repair, and angiogenesis.^28^ TGF-β_1_, an isoform expressed copiously in trophoblasts and endothelial cells, functions as an apoptotic and proliferative mediator of vascular endothelial cells, immunosuppression and production of the cellular matrix.^29^ Furthermore, TGF-β_1_ contributes to the normal placentation through the control of trophoblast invasion.^30^

However, in pre-eclampsia it affects trophoblast cell migration and influences spiral artery conversion by activating gene transcription and increasing the synthesis of matrix proteins.^31^ It also decreases pericellular proteolysis by decreased synthesis of proteolytic enzymes such as the serine and matrix metalloproteinases (MMPs), and increases the synthesis of tissue inhibitors (TIMPs), thereby modifying the repertoire of cell adhesion receptors such as the integrins.^31^ In the current study we were unable to demonstrate any significant difference for TGF-β1 between the groups.

Endoglin (Eng), a co-receptor for both TGF-β_1_ and TGF-β_1_3 is highly expressed in syncytiotrophoblasts and endothelial cells.^29^,^33^ It is identified as a pro-angiogenic factor that regulates vascular remodelling and homeostasis via the endothelial nitric oxide synthase pathway.^29^,^32^-^34^ In contrast, sEng prevents the signalling pathway of TGF-β_1_ and the endothelial stimulation of TGF-β_1_-mediated nitric oxide synthase pathway, thereby obstructing endothelial and capillary development.^31^,^35^

Consequently, the anti-angiogenic effects of sEng evident in pre-eclampsia occur via its interaction with TGF-β_1_, resulting in the inhibition of the endothelial attachment of TGF-β_1_ and the subsequent loss of the endothelial pro-angiogenic and vasodilatory effects of TGF-β_1_.^22^,^31^,^34^,^35^ Therefore the clinical significance of elevated levels of sFlt1 and sEng and their role as powerful anti-angiogenic factors through their interaction with the circulating levels of VEGF, PlGF and TGF-β_1_, respectively, is well established.

Our study confirmed previous reports of elevated serum levels of sFlt1 and sEng in pre-eclamptic compared with normotensive groups.^10^,^27^,^36^ In addition, we showed a significant difference for sFlt1, sEng and PlGF between all groups. Furthermore, sFlt1 and sEng were higher in the pre-eclamptic (HIV negative and positive) compared with normotensive pregnancies (HIV negative and positive).

In our study, the HIV-negative normotensive pregnant women had higher levels of PlGF compared with both pre-eclamptic groups, confirming previous reports.^21^,^27^,^37^ PlGF was reduced in the HIV-positive normotensive versus both the HIV-negative and HIV-positive pre-eclamptic groups. Pre-eclampsia is associated with decreased levels of PlGF, with the concurrent increase of sFlt1. This trend was observed in our study among the HIV-negative normotensive versus the pre-eclamptic groups. Unexpectedly, this trend was reversed in the HIV-positive normotensive group. It is therefore possible to assume that the immune insufficiency stimulated by HIV infection reduced a predisposition to immune hyper-reactivity, forestalling the development of pre-eclampsia. Moreover, there was a significantly higher difference between the HIV-negative and the HIV-positive normotensive groups.

The sFlt1/PlGF ratio has diagnostic predictor test value for pre-eclampsia.^38^,^39^ It is therefore evident that the clinical significance of the sFlt1/PlGF ratio, which represents the antiangiogenic role in pre-eclampsia, characterises the stability between sFlt1 and PlGF. Our results showed a lower sFlt1 and sFlt1/PlGF ratio in the HIV-positive pre-eclamptic compared with the HIV-negative pre-eclamptic groups, indicative of an apparent trend towards a diagnostic value. The imbalance that occurs in pre-eclampsia may be attributed to the immunological nature of the disease, however this requires further investigation.

Unlike sFlt1 in the HIV-positive pre-eclamptics, sEng varied compared with the HIV-negative pre-eclamptic groups. Furthermore, when combined as an anti-angiogenic ratio (sEng/TGF-β_1_), a significantly lower difference was evident between the control and pre-eclamptic groups. The HIV-negative and HIV-positive pre-eclamptic pregnant females showed higher sEng/TGF-β_1_ ratios compared with both normotensive groups.

The HIV-positive pre-eclamptics showed a higher ratio compared with the HIV-negative pre-eclamptic groups, while the average ratios of the HIV-positive normotensives were lower than the HIV-negative normotensive pregnant women. These results, albeit in term pregnancies, suggest that these ratios may have a clinical significance during early pregnancy as a predictor test for the development of pre-eclampsia.

An elevation of plasma and platelet-depleted plasma levels of TGF-β_1_ in pre-eclampsia compared with normotensive pregnancy has previously been reported.^40^ Our study, however, demonstrated no significant differences in serum TGF-β_1_ levels, with a concomitant significance of sEng/TGF-β_1_ ratio for the HIV-positive pre-eclamptics versus HIV-positive normotensives, and the HIV-negative pre-eclamptics versus HIV-positive normotensives.

Our results were similar to, albeit higher than, that observed by Huber *et al.* (2002), showing no significant difference in TGF-β_1_ expression between the pre-eclamptic and normotensive groups.^41^ Noteworthy, the analyses of sEng/TGF-β_1_ ratio in our study implicated a role for TGF-β_1_ in the pathogenesis of pre-eclampsia. However, a limitation to our study was the relatively small sample size, and this requires further investigation.

## Conclusion

Our study demonstrated elevations in both sFlt1 and sEng levels in pre-eclamptic compared with normotensive pregnancies, irrespective of the HIV status. Quantification of serum pro-angiogenic/anti-angiogenic factors in HIV-associated pre-eclampsia is novel.
